# Fatal transorbital penetrating intracranial injury caused by a bicycle hand brake

**DOI:** 10.1186/1865-1380-5-34

**Published:** 2012-09-18

**Authors:** Willemijn B Huiszoon, Pieter N Noë, Albertus Manten

**Affiliations:** 1 Department of Critical Care, Meander Medical Center, Utrechtseweg 160, Amersfoort, ES 3818, the Netherlands; 2 Department of Emergency Medicine, Meander Medical Center, Utrechtseweg 160, Amersfoort, ES 3818, the Netherlands

**Keywords:** Transorbital penetrating intracranial injury, Bicycle hand brake, Glasgow Coma Scale score

## Abstract

A transorbital penetrating intracranial injury is a rare and severe traumatic brain injury. Patients with this type of injury may present dramatically, but often the injury is subtle and therefore easily overlooked and not recognized in the first place. We present the case of a 45-year-old female admitted to the emergency department after she fell with her bike and the bicycle brake handle penetrated her left eye. A computerized tomography of the cerebrum showed a fracture of the superior orbital roof with multiple bone fragments extending into the brain near the circle of Willis. A pneumocephalus and traumatic frontobasal, intraventricular and subdural hemorrhage was seen. The patient deteriorated suddenly and was transferred to a neurosurgical center where she underwent an emergency craniotomy with evacuation of the intracerebral hematoma and an intraventricular drain was placed. After surgery, the patient’s condition deteriorated, and total compression of the brain stem occurred, upon which the patient was declared brain dead. Our case report shows that the Glasgow Coma Scale score at admission is not always a good predictor of the severity of the injury. Even when there is minimal suspicion of a penetrating intracranial injury, a computerized tomography should be performed immediately, independent of the patient’s Glasgow Coma Scale score. A direct transfer to a specialized neurosurgical center is recommended because this injury often results in death due to fatal complications such as intracerebral hemorrhage, pneumocephalus and brain stem injury.

## Case report

Transorbital penetrating intracranial injury (TPII) is rare and is considered a severe traumatic brain injury. Historically, this type of injury was frequently seen in a military setting, but over the past 20 years several civilian cases of TPII have been described [1,2]. The available literature mostly describes low-velocity TPII in children caused by a variety of foreign bodies such as pencils or scissors [3,4]. Patients with this type of injury may present dramatically, but many injuries are subtle and may be occult.

A 45-year-old female presented to our emergency department with a trauma of her left eye. She had accidentally fallen when trying to mount her bicycle. She fell on top of her bicycle, and the brake handle penetrated her left eye. She referred herself to our emergency department and initially only complained of acute vision loss and an extremely painful left eye. She had a Glasgow Coma Scale score (GCS score) of 15, and vital parameters were normal. Trauma screening only revealed an isolated injury of the left eye, which was swollen, and a periorbital hematoma was seen. The eye globe was perforated with a deformed pupil. There was complete vision loss, and movement of the left eye was impossible. Neurological examination revealed no loss of sensibility or motoric dysfunction.

**Figure 1:**
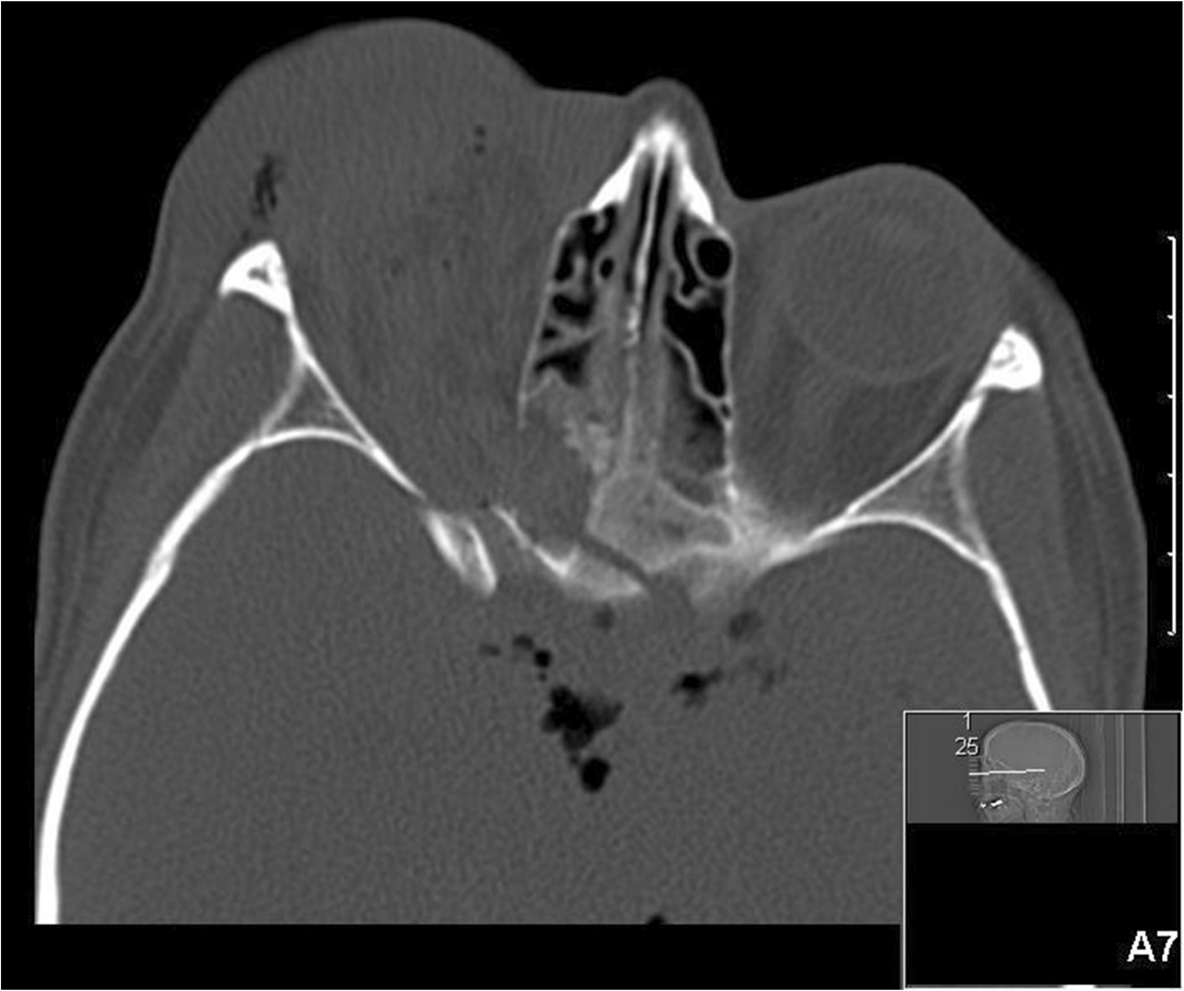
CT showing a left orbital roof fracture and pneumocephalus.

**Figure 2:**
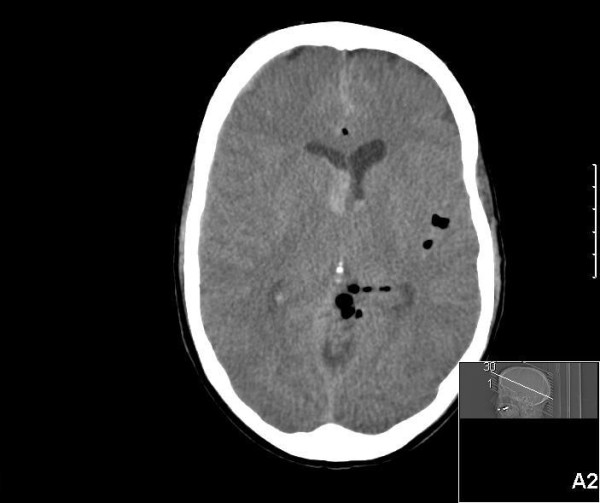
CT showing a subarachnoid and intraventricular hemorrhage and pneumocephalus.

A computerized tomography of the cerebrum was made and revealed a fracture of the superior orbital roof with multiple bone fragments extending into the brain near the circle of Willis (Figure 1). Pneumocephalus and traumatic frontobasal, intraventricular and subdural hemorrhage were seen (Figure 2). After the CT, she was prepared for emergency transfer to a nearby neurosurgical center. While waiting for the ambulance, the patient suddenly developed a generalized epileptic insult not responding to anti-epileptic medications. Eventually a status epilepticus developed, managed by intubation and sedation with propofol. During the ambulance transfer, the blood pressure increased and heart rate decreased. The right pupil became wider and did not react to light anymore. These findings were suggestive of brain herniation and brain stem compression.

Upon arrival at the neurosurgical center, the patient was taken directly to the operating room for an emergency craniotomy. Evacuation of the intracerebral hematoma was performed, and an intraventricular drain was placed. A CT, magnetic resonance imaging (MRI) and magnetic resonance angiography (MRA) were performed, which revealed an obstructive hydrocephalus and extended intracerebral hemorrhage. Despite the emergency exploration, the patient’s condition deteriorated after surgery. A total compression of the brain stem occurred, and the patient was declared brain dead, after which she was found eligible to be a heart-beating donor.

To the best of our knowledge, this is the first case report describing a transorbital penetrating intracranial injury (TPII) in an adult patient caused by a brake handle. This trauma mechanism has been described in previous reports mostly in young children [5-9].

Three reports described a fatal TPII in children who were all unconscious at the time of presentation to the emergency department [7-9]. In contrast to these reports, our patient was fully conscious when she presented to the emergency department. This shows that the GCS score at admission is not always a good predictor. It also underlines the importance of an early CT in patients with an occult injury or subtle presentation to determine the extent of the injury and detect intracranial lesions. When there is a suspicion of a non-metallic intracerebral foreign body, it is better to perform an MRI as CT misses 42% of the non-metallic foreign bodies [10].

The patient in our case report underwent an emergency neurosurgical intervention. The other reports did not describe the performance of a neurosurgical intervention in children with TPII caused by a brake handle. A possible explanation could be the low GCS score on admission and the extent of the injury associated with a worse prognosis. Overall, the literature about the effect of neurosurgical treatment in the acute management of TBII is scarce, and the prognostic effect is not clearly described.

We described an adult patient with a transorbital penetrating intracranial injury caused by a bicycle handbrake. It is important to recognize this type of injury at the time of presentation because it can lead to fatal complications such as intracerebral hemorrhage, pneumocephalus and brain herniation [11]. Despite the maximum GCS score at presentation and an emergency craniotomy, our patient did not survive.

## Abbreviations

CT: Computerized tomography; GCS score: Glasgow Coma Scale score; TPII: Transorbital penetrating intracranial injury; MRI: Magnetic resonance imaging; MRA: Magnetic resonance angiography.

## Competing interests

The authors declare that they have no competing interests.

## Authors’ contributions

PN and AM were involved in direct patient care. PN obtained verbal consent from the husband of the patient for publication of this case report. WH researched the current literature and wrote the case report. PN and AM oversaw and critically revised the manuscript. All authors read and approved the final manuscript.
